# Building a Sustainable Construction Workforce

**DOI:** 10.3390/ijerph16214202

**Published:** 2019-10-30

**Authors:** Rosemary K. Sokas, Xiuwen Sue Dong, Chris Trahan Cain

**Affiliations:** 1 School of Nursing and Health Studies, Georgetown University, Washington, DC 2005, USA; 2 CPWR–Center for Construction Research and Training, Silver Spring, MD 20910, USA; sdong@cpwr.com (X.S.D.); ccain@cpwr.com (C.T.C.)

**Keywords:** aging workforce, older workers, construction workers, labor unions, union interventions, sustainable workforce, work accommodations, career pathways

## Abstract

The average U.S. construction worker is aged 42.6 years, and will not be eligible for full Social Security retirement benefits until age 67. Delayed retirement is largely driven by economic need, but construction workers face considerable challenges in remaining on the job. This study explores trade-specific age trends within the construction industry, and the experiences of building trade unions with aging membership. A mixed-methods approach used trade-specific age statistics from the Current Population Survey and key informant interviews with labor leaders, in order to identify union experiences and interventions. Mean and median ages for all subgroups in construction increased from 2003 to 2017. Immigrant construction workers were significantly younger than workers who were born in the U.S. (41 vs. 43, *p* < 0.001). Union workers were older than non-union workers (42 vs. 39 in 2017, *p* < 0.001); the age differential between self-employed and wage-and-salary workers was wide (49 vs. 40, *p* < 0.001). Union leaders described barriers, such as age discrimination and the loss of previously available light tasks, as well as current and potential solutions through union contract language requiring the inclusion of older workers, or establishing limits for lifting. Other solutions included career pathways for training and safety, with their attendant limitations; mentoring/pairing opportunities with apprentices; and the potential opportunities and training needs for site management positions.

## 1. Introduction

The age of the average U.S. worker continues to increase; in construction, aging of the workforce has accelerated as labor markets tightened, following recovery from the Great Recession. Between 1994 and 2024, the percentage of U.S. workers aged 55 years and older is projected to double, increasing from 11.9% to 24.8% [1]. Between 2016 and 2026, the average age of the U.S. workforce is expected to increase from 42.0 to 42.3 years—“the highest level ever recorded” [2].

Loss of defined-benefit pension plans, loss of retiree health care benefits, increasing age eligibility requirements for full Social Security retirement benefits, and the impacts of the 2007 recession, have increased financial pressures to continue working [3,4,5,6]. These factors have had an even greater impact on the construction industry, which is particularly sensitive to business cycles, and has seen wide swings in employment and in workforce characteristics over the past two decades [7]. Exploring data from the Health and Retirement Survey, a nationally representative cohort of 26,000 individuals born between 1931 and 1959, Dong and others documented a decline in union membership and in defined-benefit pension plans among construction workers, as well as an increase in the proportion of workers who self-report planning to work fulltime beyond age 65, despite a general decline in self-reported health [8]. The average age of construction workers increased from 36 to 42.5 years between 1985 and 2015, and the proportion aged 55 and older increased from 12% to more than 20% [9].

Construction workers face significant challenges working at older ages, however, and those with poorer self-rated health do plan for an earlier retirement, regardless of financial circumstances [9]. Work in physically demanding jobs has been associated with increased disability and premature mortality [10]. Construction remains among the most physically demanding and hazardous industries, with fatal and non-fatal traumatic injury rates triple those for all industries [11]; construction workers are more likely to have sustained work-related injuries over time that limit functional capacity [12].

The burden of prior work injuries negatively impacts the construction workforce as it ages. Using the National Longitudinal Youth Survey, a nationally representative longitudinal survey initiated in 1979, Dong and others found significantly more workplace injuries, including both injuries resulting in days away from work (DAFW), and all injuries among construction workers, compared to other workers, as expected. In the year following a workplace injury, construction workers were significantly more likely to have lost wages, worked fewer hours, or been laid off; they were significantly less likely to have been offered modified work [13]. Comprehensive health surveys were conducted when participants reached age 40, by which time 38% of the construction workers had experienced a DAFW injury. When compared with construction workers who had not been injured, those with previous DAFW injuries were significantly more likely to report diagnosed conditions, musculoskeletal disorders, depression and health problems limiting ability to work or to accomplish other tasks, such as climbing stairs, an average of 10 years after the injury had occurred [12]. Scott and others found that Canadian workers who sustained permanent work-related injuries were more likely than others to exit the workforce; low-wage workers and those in physically demanding jobs were at increased risk of exiting [14]. A follow-up study of disability retirement among Swedish construction workers found much higher rates among those with physically challenging jobs, such as rock workers and concrete workers, compared to electricians and foremen [15]. In a study of U.S. construction workers between the ages of 40 and 59 years, Welch et al. identified musculoskeletal disorders and other medical conditions to be associated with a premature exit from the workforce [16]. In addition to chronic musculoskeletal disorders, older construction workers have very high rates of noise-induced hearing loss, as well as exposure to solvents, lead and a variety of respiratory hazards, leading to chronic neurologic and respiratory disorders [17,18,19,20,21,22,23]. Depression and mental health disorders associated with prior work-related injury also predict premature workplace exit [24]. Across all industries, health may mediate work-related stressors associated with the ability to perform tasks [25], while periods of involuntary unemployment, which are common in construction, have been associated with adverse cardiovascular outcomes [26].

Injury severity also increases with age. The number of days lost per DAFW injury begins to climb at age 35, and is ten times higher for workers over the age of 65 compared to those under 19 [11]. The rate of fatal traumatic injuries for workers 55 and older is twice that for workers under 35, and this increased fatality rate is true for most categories of injury, including struck-by, falls and transportation [27,28,29].

While construction workers experience increased disability from past work exposures and sustain greater impacts from newly-acquired injuries, they find few opportunities for modified duty as they attempt to continue to work [30]. In addition to high physical demands and high injury rates, construction work is characterized by constantly changing work environments, long commutes, interrupted employment, small contractors and non-standard working arrangements. Both the 2004 National Academies of Science report and a 2009 “Healthy Aging for a Sustainable Workforce” conference report advocated for primary prevention through the enforcement of existing safety and health regulations for all workers, additional research into work modifications that could address ergonomic and other hazards, and more targeted policies to provide supports to older workers specifically [31,32]. In the Netherlands, Tonnon and colleagues have explored concerns about sustainable employability in the construction industry among both workers and employers [33,34]. However, although the challenges to older workers in the construction industry have been well documented, interventions, where they exist, remain scattered and piecemeal.

In the U.S., the unionized construction industry accounts for under 20% of the production construction workforce, and has been characterized by improved economic and health outcomes and approaches to job security [35,36]. As such, union approaches may offer positive examples that might be adapted to scale. In construction, the individual crafts are represented by distinct unions. The aim of this study was twofold: To characterize the age structure in specific construction trades, and to explore how representatives of the 15 U.S. building trades unions and affiliated organizations perceive the experiences of older workers, describe solutions that have been developed, and offer recommendations for addressing unmet needs.

## 2. Methods

This study included quantitative exploration of nationally representative data in the U.S. and qualitative data from construction trade union leaders. This combined method allows us to examine how the general trend of an aging workforce affects specific segments of the construction industry, and how the different craft trades experience and respond to these trends. Human subjects’ protection review and approval was provided by the CPWR Institutional Review Board.

### 2.1. Quantitative Methods

Age statistics for construction workers were obtained from the Current Population Survey (CPS). The CPS is the primary source of labor force statistics for the population of the United States. It is a monthly household survey conducted by the U.S. Census Bureau for the Bureau of Labor Statistics (BLS). The CPS collects basic demographic data from a scientific sample of about 60,000 households, including age, gender, race and Hispanic origin. For those aged 16 years or older, the survey collects detailed information on employment, such as occupation, industry and hours worked. To increase data reliability, three years of data from 2015–2017 CPS were pooled together to estimate the impact of immigrants upon the aging workforce. Means and median of age and the 95% CI were reported for each selected occupation. T-Test was used to measure whether the difference in age between native-born and immigrants by occupations in construction was statistically significant at a *α* = 0.05 level. SAS survey procedures were applied for all analyses using the CPS data [37].

### 2.2. Qualitative Methods

The 14 unions comprising North America’s Building Trades Unions (NABTU) each have occupational safety and health directors who meet monthly to discuss occupational safety and health (OSH) concerns [38]. The Center for Construction Training and Research (CPWR), a non-profit research and training organization focused on occupational safety and health in the construction industry, leads and supports these meetings. Following a brief presentation and review of demographic and occupational safety and health information concerning the aging workforce to structure the problem, committee discussion generated the background for semi-structured key informant interview questions [39]. Open-ended questions were designed to elicit opinions and suggestions about whether and how the participant experienced aging as a factor in the trade; whether accommodations or contract language were available for workers with physical impairments; approaches to mitigating physical demands on the worksite or to create career pathways with less physical demands; and any other needs identified by the interviewee. Draft questions were adapted following feedback from the NABTU OSH committee members. CPWR leadership and members of the NABTU OSH committee nominated key informants, who were each contacted using Institutional Review Board (IRB)-approved outreach materials. In addition to the NABTU-affiliated unions, an additional large construction union not affiliated with NABTU also participated in this study. Several participants brought colleagues into the telephone interview, and others suggested additional participants from their union who could provide further perspectives. Key informants were recruited among leadership in the union organizations, while the interviews were conducted by a researcher outside of the industry structure, minimizing the risk of influence in responses to open ended questions.

One interviewer conducted and transcribed the interviews. Two investigators initially open coded the transcripts independently, using grounded theory and subsequently discussed and reviewed results to identify themes. On all major themes, saturation appeared to have been reached. However, one important theme included the differences inherent in the different craft trades. To maximize information capture, all examples of interventions were tabulated.

Preliminary results were presented to the interviewees at a regularly scheduled meeting of the NABTU OSH committee to identify errors or gaps in the information captured. Subsequent written and verbal input from one of the committee members was incorporated into the final analysis.

## 3. Results

### 3.1. Quantitative Results

**Figure 1:**
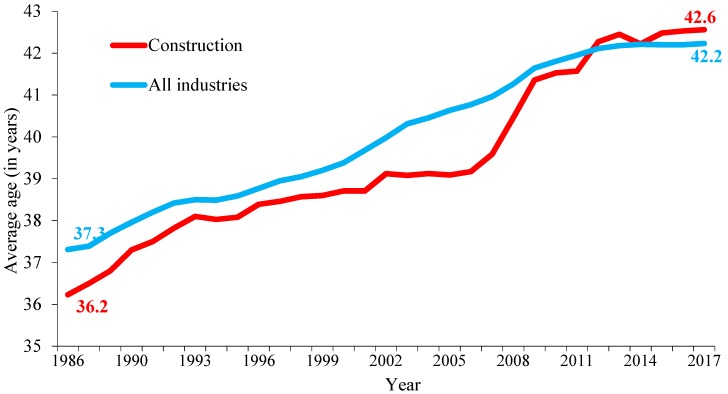
Average age of workers, construction versus all industry (all employment).

Although construction workers historically have been younger than the overall workforce in the U.S., they are aging rapidly. In 2017, the average age of construction workers reached 42.6, exceeding the average age (42.2 years) of the U.S. workers, 3.5 years older than that in 2003 and 6.4 years older than three decades ago (Figure 1).

Mean and median ages for workers in all occupations in construction increased between 2003 and 2017. Mean age for most of selected occupations exceeded 40 years except helpers, roofers and construction laborers (Table 1). Immigration reduces the age of the workforce, as expected. Without immigrant workers, the U.S. construction workforce would average about a year older. Immigrant effects varied among occupations (Table 1).

**Figure 2:**
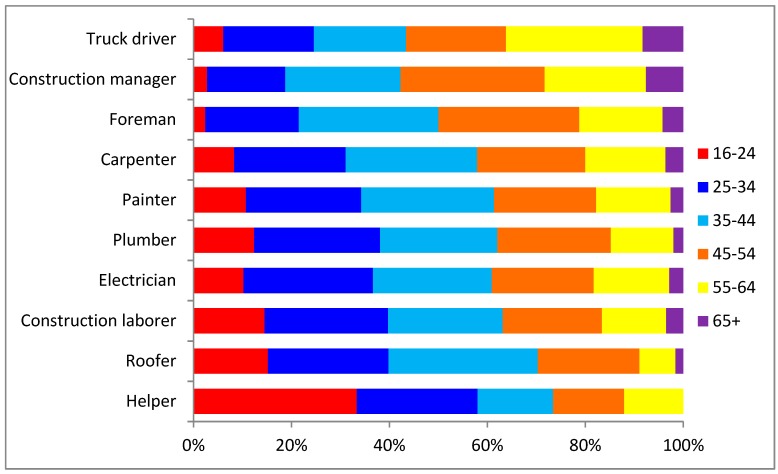
Age distribution among selected occupations in construction, 2017 (All employment).

**Figure 3:**
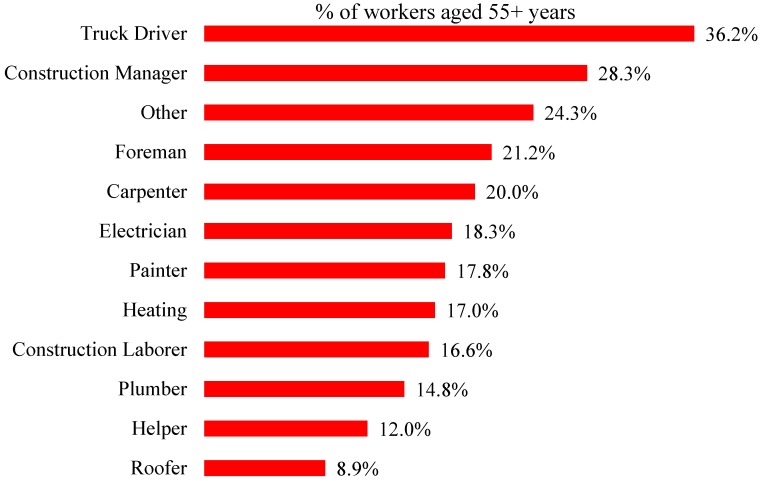
Percentage of construction workers aged 55+ years, selected construction occupations, 2017 (All employment).

In 2017, the proportion of truck drivers, construction managers and foremen aged 65 and older exceeded the proportion aged under 25 (Figure 2). For all construction, one in five workers is aged 55 or older, with administrative support personnel and truck drivers tied at 28.8% in this age category (Figure 3).

**Figure 4:**
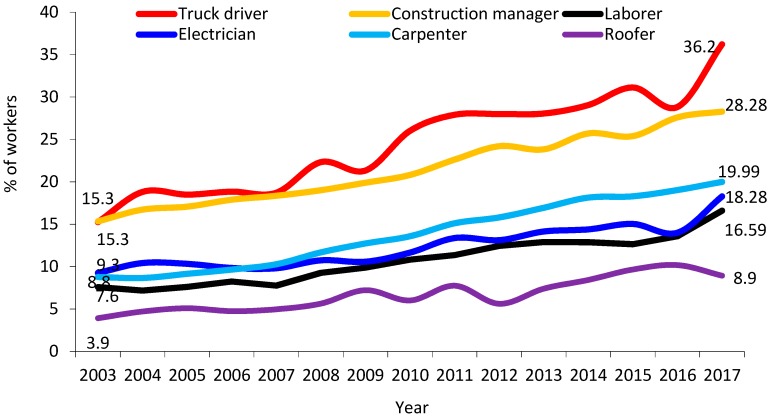
Construction workers aged 55+ years, selected construction occupations, 2003–2016 (All employment). Source: Current Population Survey, 2013–2017, Calculations by the authors.

The proportion of workers age 55 and older increased in all occupations, including roofers and laborers, between 2003 and 2017 (see Figure 4).

Age varied among worker subgroups. Self-employed workers were much older than wage and salary workers, with the median age at 49 (self-employed) and 40 (wage) in 2017, respectively (*p* < 0.001). In 2017, the median age for union members was 42 years, compared with 39 years for non-union workers (*p* < 0.001). Similarly, the average age was significantly different between union and nonunion workers for most occupations, except helpers (Table 2).

### 3.2. Qualitative Results

Seventeen representatives from 13 building trades unions participated in the semi-structured interviews. Additionally, one NABTU official and one construction management university program director provided full interviews, for a total of 19 key informant interviews. Another union official contributed additional information by email and telephone following the preliminary presentation of the results. The two unions that did not provide formal interviews offered informal verbal and email information (see Table 3).

The majority of the key informants had been in the trade before assuming leadership positions, while a substantial minority had technical safety and health, research, or economics backgrounds. Of the 12 key informants who started in the trade, the average length of union membership was nearly 33 years, ranging from 20 to 49 years. Most had explicit responsibility for workplace safety and health, but titles and responsibilities varied, including Director of Safety and Health, International Vice President, Director of Benefits, Director of Research, Director of Apprenticeship and Training Programs, Trust Administrator, Executive Director, Research and Education Joint Trust Fund, Director of the HazMat Training Program, Technical Coordinator and Executive Director, Training Fund. Key informants had held their current positions for an average of almost eight years, with a range of one to 21 years. Two participants were African American, and two were female, all others were white males.

#### 3.2.1. Aging Workers

When asked whether aging was a factor in their trades, it was clear that within construction, the individual trades differed in important ways. All trades impose physical challenges, but there is a range of effort required for various tasks. The structure of pension funds influenced how long typical workers worked in the individual trades. The pension funds varied—in some unions, a member can retire with adequate income and health benefits after 30 years on the job, and most members choose to do so. This “30 and out” ability means that some trades have few members over 60. As one respondent put it, “the aging workforce is getting younger” (because of improvements in pensions over time). In others, however, members as old as 70 have not accrued sufficient pension benefits and “don’t have the hours to retire”. Most respondents agreed that those who can afford to retire do so, resulting in a skilled trade shortage in many areas. New worker recruitment challenges are common in most (but not all) trades, exacerbating the shortage of workers.

Reports varied about what older workers experience in the trades. On the one hand, the shortage of skilled craft workers favors the retention of older workers. According to one respondent, contractors value an older worker with skills, particularly if the job is technically challenging on a tight timeline. This experience often includes safety awareness: “Older workers have seen what can go wrong and pay more attention—they have seen friends injured, and know someone who died”.

On the other hand, increased workers’ compensation costs for older workers have resulted in contractors laying off older workers. Many noted that overt age discrimination is “hard to prove”, and that there is generally a negative attitude on the part of workers as well as employers towards asking for help. If a task requires a lower skill level and a there is “a lot of ground to cover”, employers will choose cheaper apprentices, hiring several younger workers and laying off older ones. Several respondents reported that “Contractors want to hire 25 year-old kids with 25 years of experience.” Respondents raised concerns that fitness for duty testing—lifting/crawling/range of motion requirements—were deliberately targeting the many older workers who cannot meet all the requirements: “It’s a way to weed out hurt workers.”

Respondents attributed the early retirement of those who could afford it to the wear and tear experienced by years in construction work—“old age begins at 40”. Virtually all interviewees offered descriptions of the impact of the trade on older workers’ long-term health. Respondents from different trades specified the following types and areas of injury: Backs (many having had surgery); knees/backs/hips (from heavy equipment/vibration); knees (from crawling); backs, knees, hips, shoulders, carpal tunnel syndrome; back/knees/shoulders; asbestos-related diseases; neuropathy (from solvents).

Additional challenges vary by trade, and generally include weather, work organization, travel times/extended work hours and new technology that increases work challenges. In some trades, 100% of the work is conducted outdoors; for one trade, a respondent described the work as having to be done in batches after other trades have been through, making it more difficult to gain access to the worksite or to use lifting or other assistive devices. Ten-hour workdays and six-day weeks are common, and for some projects workers either commute up to 100 miles on top of long workdays, or are away from home for prolonged periods, living in trailers. One respondent described a transformative technology in the form of additives that allow quicker pour, but still require hand finishing, so that jobs that previously took 10–12 craftsmen are now handled with 3–4, who experience increased pace and ergonomic stressors.

#### 3.2.2. Work Accommodations

When asked about work accommodations for short-term or long-term impairments (including age-related), most respondents reported few or no return-to-work accommodations except those that reduce Workers’ Compensation costs (including punitive make-work): “There is no light duty.” Respondents with many years in the trade provided accommodation examples from decades ago, including an older worker who had rebar brought to him, where he could mark the center and perform layout; a number of examples in which older workers were teamed with younger workers; and work as a “walking supervisor”. 

Previous opportunities for light work are reported to no longer exist in the industry. Recent accommodation examples generally addressed short term needs, and included work in a fabrication shop that was still heavy work but required no climbing; maintaining and monitoring tools in a tool room; fire watch/hole watch/confined space watch; work on a saw requiring less overhead/layout/set up; driving the regular car ahead of a heavy vehicle on a highway; site traffic control, opening or securing a site, garbage control, clean up, ordering materials.

#### 3.2.3. How Unions Responded

##### Collective Bargaining Agreements (CBAs)

Most unions include standard prohibitions against age discrimination in clauses prohibiting various forms of discrimination in their CBAs, but do not include ratios. In response to concerns about discriminatory practices, one union has included the following contract language about age ratios in several of its contracts: “For any shop employing five (5) or more journey workers, if available, at least every fifth (5th) journey worker shall be fifty (50) years of age or older.”

CBAs can also address priary safety and health prevention practices through language addressing lifting limits, such as requiring two persons to lift loads greater than 50 lbs., or requiring OSHA 10 or OSHA 30-hour hazard awareness training, to include fall prevention, confined space and ergonomics. CBAs may ensure that the employer continues to pay into Health and Welfare and pension funds for injured workers. Although not formally part of the contract process, many respondents described union representatives negotiating accommodations for individual members.

##### Mentoring/Pairing

Many respondents recognized a need for mentorship programs, although these were largely driven by the challenges many of the trades face with recruitment: “If we don’t do something, we won’t have the people.” While not primarily aimed at assisting the older worker, such assistance is an anticipated benefit. Challenges arise from intergenerational distrust, including reported comments from older workers such as “Millennials don’t care about older workers.” and “I’m not teaching them what I know, they’ll take my job.” There is also a long-standing culture in construction that is seen as a problem in need of fixing: “In the old days, [we] got yelled, screamed and hollered at—can’t do that anymore.” Opportunities focus on making the pairing a two-way street, with older workers paired with apprentices to mentor, and younger workers helping with physically demanding tasks. One union has launched a mentorship program for culture change that focuses on diversity, recruitment and retention that includes training for mentors and pairs apprentices with journey workers on job sites.

##### Career Pathways

Despite long standing opportunities for experienced workers to develop skills to become apprenticeship trainers or safety practitioners, these opportunities were not seen as viable solutions to address the scale of the problem since the number of positions is limited. Furthermore, many of the new approaches for just-in-time construction require advanced computer skills. Union-sponsored training programs for safety, training, contracting, etc., may lead to careers outside the trade. In some specialized trades, union members may start their own small companies, although this typically does not reduce their workload. One approach encouraged by NABTU and by many of the individual unions is training and education programs that vary from course work leading to certificates, to degree-granting programs to enable workers to take jobs that are less physically demanding. “Older workers want to get into the trailer,” and money is not the reason. Trades differ in the types of jobs workers may move into, such as foremen or supervisor (depending on the trade, this may not reduce physical demands); purchasing agents, business agents, or consultants for “pre-planning” site safety supervisors, inspectors, safety trainers or apprenticeship trainers. Formal educational programs tend to focus on construction management, taking advantage of prior experience and skills, and in many instances, utilizing academic credit from prior apprenticeship programs. 

Several of these programs pair with local community colleges. One national program, supported by partial scholarships from NABTU, offers a four-year degree in construction management through an online program at Rowan University. However, a number of respondents noted the challenges workers face, given long work hours and travel times that make participation even in online classes difficult.

One union has developed an innovative formal second apprenticeship program, recently approved by the Department of Labor, that is a two-year program for journey workers who train to become inspectors to meet the requirements for bridge and other structural painting. As a second apprenticeship, the program entails a modest income decrease temporarily, but offers a sustainable career that utilizes the prior skills of the trade enhanced to certify the worker to conduct formal structural safety inspections.

#### 3.2.4. Other Needs Identified

Respondents differed on the need for more research. In some trades, assistive technology is already widely available—“if an employer is not using it, [he] doesn’t want to.” The respondent suggested research demonstrating that the Workers’ Compensation rate impact could be useful. Others emphasized the importance of planning: “So what if you can’t climb towers, there are a million other things you can do;” “Play the quarterback as a quarterback.” One respondent suggested simple solutions, such as placing a forklift in each truck delivering materials to a site, to be returned with the truck.

In other trades, respondents identified the need for new interventions, but expressed concerns about feasibility. Several mentioned exoskeletons as promising, but expensive; others noted the need for improved robotic assistive devices or mechanical lifting assists.

#### 3.2.5. Other Topics Raised

Other topics that emerged included comment about the positive role manufacturers are playing by developing and disseminating equipment and information to reduce ergonomic strain, as well as the positive role some larger employers play by implementing lifting and other ergonomic interventions.

Recruitment emerged as an important related issue. As noted above, many skilled trades are facing a wave of boomer retirements. Respondents attributed difficulty recruiting apprentices to a number of factors, ranging from the demise of high school shop classes, to what participants perceived to be an unrealistic emphasis on college, which many start but do not finish. They noted that, on the other hand, others go into construction without apprenticeship training or union membership, not realizing what pension or other benefits that they will miss. Most reported that apprentices are entering the trades at older ages. One union has initiated a Recruitment and Retention Task Force to attract and keep non-traditional students and veterans.

## 4. Discussion

Construction workers in the U.S. are aging rapidly in all trades and occupations. While immigrants lower the average age of the workforce, even they are aging: The average age of immigrant construction workers is now 40.1 years. One in five construction workers is over the age of 55, with truck drivers and construction managers and foremen among the oldest. Skilled union members are older than non-union members by an average of over two years, and the average age for union laborers, plumbers, carpenters, painters, roofers and electricians exceeds 40 years.

The union leaders interviewed for this project had many years of experience and directly lived the historical trends affecting retirement in the U.S., having apprenticed at a time when older members of the trade (who had begun work prior to World War II) did not have robust pension plans, and working past age 65 was more common. 

They witnessed the decline in strategies to maintain this older workforce, such as jobs tracking tools, or the routine matching of apprentices to older workers in mentorship relationships that allowed the older worker to rely on the younger apprentice to do the more physically demanding jobs. They universally acknowledged the disappearance of these arrangements over time as work and productivity demands increased.

Key informant interviews highlighted the extent to which the current labor shortage, particularly in skilled workers, is being felt across the unionized construction industry and beyond. This finding has been extensively documented, has been associated with a loss of productivity and schedule overruns in the industry [40], and has sparked interest in worker recruitment and retention [41]. Many of the participants in this study saw the challenges related to developing appropriate accommodations for aging workers as intertwined with challenges recruiting apprentices in their trades, some raising concerns about being able to maintain union jobs if they are unable to field the numbers of skilled workers needed. The innovations undertaken by several unions include explicitly training experienced workers to function as supportive mentors to enhance the recruitment, diversity and retention of apprentices. The intervention offers the potential to enhance the role of older workers, while improving worksite culture to make the trades more appealing to a more diverse workforce. Providing older workers with physical assistance in a form that would be perceived as acceptable is an anticipated by-product of such mentorship pairing. These findings echo those which Truxillo and others identified in a review of issues concerning aging workers in all industries, reflecting the increased focus on generativity as individuals age, as well as the need for ergonomic interventions for workers of all ages [42].

Pre-retirement exposure to heavy physical demands and environmental hazards has been associated with worsened pre- and post-retirement health status [43]. High physical demands, safety and health hazards, and prolonged workhours and associated travel, are identified not only as producing disability among older workers and preventing workers with impairments from continuing to work, but also as deterrents to recruiting new workers. These perceptions echo those of Dutch blue collar construction workers surveyed about sustainable employability, who ranked highly the need to reduce physical job demands [34]. Although this was also frequently mentioned by a parallel survey of Dutch construction employers, only one in five had actually listed reducing physical workloads as being “on the company agenda”, citing cost, time and client demands as barriers [33]. Interventions aimed at planning, administrative controls and engineering assistive devices, along with improved approaches to temporary as well as permanent workplace accommodations, are widely seen to benefit workers entering the trades, as well as those attempting to continue to work at older ages. Important innovations for a variety of construction tasks have been demonstrated to reduce the ergonomic stressors prevalent in many construction tasks [44,45,46]. Furthermore, key informant interviews conducted among 80 British construction trades workers identified over 250 suggestions for work and equipment modifications to reduce ergonomic stressors [47]. Participants in our study differed by trade in their assessment of the availability of adequate interventions or the need to develop new ones, although there was general agreement that only the larger employers currently implement the available interventions. While continued intervention effectiveness studies are needed, dissemination research is also needed, particularly among small and medium sized enterprises, to ensure the uptake of interventions shown to have impact.

As previously found among blue collar construction workers and construction employers [33,34], participants identified opportunities for career pathways to enable construction workers to reduce physical demands. Both individual unions and NABTU offer an array of continuing education and training programs to foster the acquisition of management, training, or safety skills. Participants from many of the trades expressed reservations about whether such pathways were scalable, however, and identified long work hours and cost as barriers to a wider uptake. One participant described an innovative project that formalizes a second apprenticeship to produce safety inspectors, which provides pay (although at a reduced rate) throughout the formal training period and fully utilizes prior experience. 

Formal assessment to determine whether similar approaches would be workable in other trades, or to evaluate programs and barriers for expansion or program modification, may be needed to improve uptake and maximize job placement.

Although study participants clearly articulated the skills and value provided by older workers, they uniformly identified age discrimination as a factor in construction worksites. Others have demonstrated the adverse impact such discrimination can have upon mental and physical health [42,48]. Union collective bargaining agreements improve the otherwise at-will employment arrangements of the private construction work sector, and union representatives can address overt instances of discrimination. However, participants overwhelmingly felt that such discrimination was hard to prove, and only one provided an example of explicit ratios for hiring older workers in contract language to attempt to prevent discriminatory hiring. Formal approaches to reducing workplace age discrimination are needed concurrent with efforts to address ergonomic and work organization challenges.

Given the shared experience of aging populations in virtually all developed countries, the themes that emerged in this study may be widely applicable. These emphasize the need to reduce physical strain for workers entering the trade, as well as for older workers; the need to protect older workers from discrimination and to provide alternative career pathways; and the need to welcome previously excluded groups, such as racial and ethnic minorities (including immigrants and refugees) and women into the construction industry. While no discussion of population age structure can be complete without addressing the importance of the global immigrant and refugee population, such a discussion is outside the scope of this report.

This study has a number of limitations. Sampling and non-sampling errors could exist in the quantitative data from the CPS, particularly for small construction occupations. While combining data from multiple years might increase data reliability, it could be problematic if year-to-year changes are taking place within that specific occupation. The qualitative findings of this study are based on the experiences of the minority of U.S. construction workers who are members of unions, and so these findings understate the extent of the problems that non-union construction workers face as they age. Furthermore, this segment of the workforce is much more likely to have defined pension benefits, including health benefits, that provide a safety net for “early” retirement at age 62 (with Medicare benefits beginning at age 65). As the age for full Social Security benefits increases to age 67 (with increased benefits for those filing at age 70, effectively penalizing those retiring before this age), and the penalties increase for those taking benefits at age 62, non-union construction workers face the reduced benefits of a frayed financial safety net. Among immigrant construction workers, those who are undocumented have no access to social security benefits (whether from failure to pay in or from inability to collect what was paid in), and have no financial safety net at all.

## 5. Conclusions

The extent and pace of aging in the U.S. construction workforce requires urgent attention to improving the safety and health characteristics of the industry to foster healthy work trajectories, reduce injuries and illnesses leading to premature aging, and prevent fatal and non-fatal injuries among older workers. Dissemination of currently available safety and health preventive measures to small and mid-size employers may require changes in the organization of work, as well as improved workplace standards and enforcement. Finally, steps to address the current shortage of skilled workers should include both approaches to keep older workers functioning safely and to welcome a more diverse workforce. Efforts to enhance mentorship training to encourage recruitment into the building trades and to build sustainable career pathways are underway, but require evaluation and dissemination.

## Figures and Tables

**Table 1:** Average age of construction workers by occupation, U.S. born versus immigrant worker, average of 2015–2017 (All employment).

	U.S. Born			Immigrants			*p*-Value *
Occupation	Mean	95% CI		Mean	95% CI		
Construction Manager	46.8	46.2	47.3	44.6	43.3	46.0	<0.0001
Foreman	45.4	44.8	46.0	44.2	42.8	45.6	<0.05
Carpenter	42.0	42.5	43.5	40.9	40.2	41.6	<0.0001
Construction Laborer	38.6	38.1	39.2	40.1	39.6	40.6	<0.01
Electrician	40.2	39.5	41.0	42.1	40.6	43.7	<0.05
Painter	43.5	42.5	44.5	39.2	38.4	40.1	<0.0001
Plumber	41.4	40.6	42.2	41.4	39.8	42.9	>0.05
Roofer	37.2	35.8	38.6	37.7	36.6	38.9	>0.05
Helper	30.4	27.9	32.9	36.3	32.8	39.9	>0.05
Heating	40.0	39.1	40.9	40.8	38.5	43.0	>0.05
Truck Driver	46.2	44.9	47.6	45.2	42.7	47.7	>0.05
Other	44.2	43.9	44.48	42.5	42.0	43.1	<0.0001
Total	43.0	42.8	43.2	41.1	40.9	41.4	<0.0001

* *p*-value for age difference between U.S. born and immigrant workers.

**Table 3:** Sources of qualitative information.

Key Informant Interview Participants	Informal E-Mail Information from Additional Sources	Safety and Health Committee Input
17 union representatives from 13 individual craft trades	E-mail responses from two union representatives declining full interviews	Preliminary presentation of issue and requires for information
One representative from NABTU as an organization	One email response from a NABTU representative	Review of and input into script/question development.
One university program director (online program)	E-mail and telephone follow up from participant in second Committee discussion	Presentation of preliminary findings—“Did we get it right? Did we get it all?”

**Table 2:** Average age of construction workers by occupation, union versus non-union, average of 2015–2017 (wage-and-salary workers).

	Union			Non-Union			*p*-Value *
Occupation	Mean	95% CI		Mean	95% CI		
Foreman	46.5	45.2	47.8	43.9	43.2	44.6	<0.0001
Construction Manager	45.1	42.9	47.3	44.7	44.0	45.4	<0.0001
Truck Driver	45.1	42.0	48.1	45.7	44.4	47.0	0.01
Construction Laborer	41.7	40.4	42.9	37.4	37.0	37.8	<0.0001
Plumber	41.1	39.7	42.6	39.3	38.4	40.2	<0.0001
Carpenter	41.0	39.8	42.2	39.0	38.4	39.5	<0.0001
Painter	41.0	37.9	44.0	38.7	37.9	39.5	<0.0001
Roofer	40.6	37.4	43.9	36.1	35.1	37.1	<0.0001
Electrician	40.1	38.9	41.3	38.7	37.8	39.5	<0.0001
Heating	37.5	35.4	39.6	38.4	37.5	39.4	<0.0001
Helper	34.9	27.5	42.3	32.2	30.0	34.4	>0.05
Other	44.0	43.3	44.7	42.2	41.9	42.5	<0.0001
Total	42.5	42.0	42.9	40.5	40.3	40.7	<0.0001

* *p*-value for age difference between union and nonunion workers.
